# Large-scale multivariate dataset on the characterization of microbiota diversity, microbial growth dynamics, metabolic spoilage volatilome and sensorial profiles of two industrially produced meat products subjected to changes in lactate concentration and packaging atmosphere

**DOI:** 10.1016/j.dib.2020.105453

**Published:** 2020-03-20

**Authors:** Simon Poirier, Ngoc-Du Martin Luong, Valérie Anthoine, Sandrine Guillou, Jeanne-Marie Membré, Nicolas Moriceau, Sandrine Rezé, Monique Zagorec, Carole Feurer, Bastien Frémaux, Sabine Jeuge, Emeline Robieu, Marie Champomier-Vergès, Gwendoline Coeuret, Emilie Cauchie, Georges Daube, Nicolas Korsak, Louis Coroller, Noémie Desriac, Marie-Hélène Desmonts, Rodérick Gohier, Dalal Werner, Valentin Loux, Olivier Rué, Marie-Hélène Dohollou, Tatiana Defosse, Stéphane Chaillou

**Affiliations:** a INRAE, AgroParisTech, Micalis Institute, Université Paris-Saclay, 78350, Jouy-en-Josas, France; b INRAE, UMR1014 Secalim, Oniris, Nantes, France; c IFIP-Institut du Porc, Maisons-Alfort et Le Rheu, France; d Veterinary Medicine Faculty, Food Sciences, Université de Liège, FARAH, 4000 Liège, Belgium; e Université de Bretagne Occidentale, EA3882 Lubem, Quimper, France; f Aerial, 67412 Illkirch, France; g INRAE, MaIAGE, Université Paris-Saclay, 78350 Jouy-en-Josas, France; h Cooperl, 22403 Lamballe, Armor, France; i Cellule Recherche et Innovation, Groupe LDC, Sablé sur Sarthe, France; j INRAE, BioinfOmics, MIGALE bioinformatics facility, Université Paris-Saclay, 78350 Jouy-en-Josas, France

**Keywords:** Meat spoilage, Food microbiota, Microbial ecology, Metabolomic, Metagenetic

## Abstract

Data in this article provide detailed information on the diversity of bacterial communities present on 576 samples of raw pork or poultry sausages produced industrially in 2017. Bacterial growth dynamics and diversity were monitored throughout the refrigerated storage period to estimate the impact of packaging atmosphere and the use of potassium lactate as chemical preservative. The data include several types of analysis aiming at providing a comprehensive microbial ecology of spoilage during storage and how the process parameters do influence this phenomenon. The analysis includes: the gas content in packaging, pH, chromametric measurements, plate counts (total mesophilic aerobic flora and lactic acid bacteria), sensorial properties of the products, meta-metabolomic quantification of volatile organic compounds and bacterial community metagenetic analysis. Bacterial diversity was monitored using two types of amplicon sequencing (16S rRNA and GyrB encoding genes) at different time points for the different conditions (576 samples for gyrB and 436 samples for 16S rDNA). Sequencing data were generated by using Illumina MiSeq. The sequencing data have been deposited in the bioproject PRJNA522361. Samples accession numbers vary from SAMN10964863 to SAMN10965438 for gyrB amplicon and from SAMN10970131 to SAMN10970566 for 16S.

**Specifications table**

**Table:** 

Subject area	Applied Microbiology and Biotechnology
More specific subject area	Microbial ecology of food spoilage during industrial production.
Type of data	Table, figure, raw sequencing data
How data was acquired	Total aerobic mesophilic population was estimated on Plate Count Agar (PCA) (Oxoid, France) for pork, (Biomerieux, France) for poultry and on de Man, Rogosa, and Sharpe (MRS) agar (Oxoid, France) for pork, (Biomerieux, France) for poultry. Both media were incubated aerobically for 48 h at 30 °C to estimate the bacterial population size in CFU.*g*^−1^ of meat. Gas composition of the trays was assessed for all trays with CheckMate3 (Dansensor, France) or Oxybaby O_2_/CO_2_ analyzer (WITT, Germany) for pork and poultry sausages, respectively. The pH of sausages was measured using FiveGo FG2 with the electrode LE427-S7 (Mettler toledo, USA). Color (L*, a*, b*) was assessed using the Minolta CR400 ChromaMeter (Grosseron, France) in CIE-Lab scale. Volatile organic compounds (VOC) were determined by HS–GC–MS on a Varian 450 gas chromatograph (Varian, USA) coupled to a Varian 225-IT mass spectrometer (Varian), equipped with a CTC Combi PAL (CTC Analytics AG, Switzerland). Quantitative descriptive analysis (QDA) was performed using seven olfactory attributes by a trained sensory panel of 15 experts. DNA amplicon sequencing was carried out with Illumina MiSeq.
Data format	Raw, analyzed
Parameters for data collection	The type of meat used for production of sausage was the first parameter analyzed (raw pork sausages and raw poultry sausages). The second parameter was the time of storage from the raw meat material up to sausages at the end of storage (primary cuts, gut casing, spices and fat). The third parameter was meat batch variability along ten sampling campaigns which were conducted for each meat product. Finally, we analyzed two process parameters: one was the influence of three doses of potassium lactate (complete dose, half dose or zero dose) used as preservative as well as three packaging atmospheres (air, and two modified atmospheres i.e. 70%O_2_/30%CO_2_ and 50%N_2_/50%CO_2_,).
Description of data collection	Meat samples were collected directly in two independent factories in France, just out of the production line. The large-scale sampling strategy was organized over a period of six months from July to December 2017.
Data source location	Samples related to pork sausages were collected in Lamballe (France, latitude: 48.4667, longitude: −2.5167) while samples related to poultry sausages were collected in Sablé-sur-Sarthe (France, latitude: 47.8376, longitude: −0.3329).
Data accessibility	Data are available as supplementary tables (Excel spreadsheets) at the institutional INRAE data repository: https://doi.org/10.15454/UDQLGE. The sequencing data have been deposited in the bioproject PRJNA522361.

## Value of the data

• The data provide a link between meat spoilage (bacterial counts, pH, color, volatile metabolic spoilage compounds non-targeted quantification further referred as “volatilome” and sensorial profiles), the packaging atmosphere, and the use of lactate and microbiota composition.
• Sequencing data can be used to understand the variation of bacterial community dynamics, abundance and diversity in these two meat products according to the type of meat and packaging atmosphere, and the use of lactate.
• Sequencing data can be used to identify biomarkers of spoilage. Accessibility to 16S rDNA and gyrB OTU (Operational Taxonomic Unit) data and detailed associated metadata allows researchers to perform new analyses with their own research purposes.
• Ten independent sampling campaigns were conducted for two meat products. A wide number of conditions were tested (three lactate concentrations and three packaging atmospheres). This large-scale sampling strategy in which over 550 samples were sequenced, at four different time points (from day 0 to day 22 of storage) provides a unique dataset for powerful statistical analysis of process parameters influencing raw meat sausage spoilage.

## 1 Data description

In the EU 20% of the initial meat production is lost, more than half occurring at animal production, slaughtering, processing and distribution steps [1,2]. This crucial economic and environmental issue in food industry is in part attributed to spoilage during storage, which is the consequence of bacterial growth and subsequent metabolic activities causing organoleptic changes of the final product unacceptable for human consumption (defects in texture, color, odor, taste or aspect) [3].

**Fig. 1:**
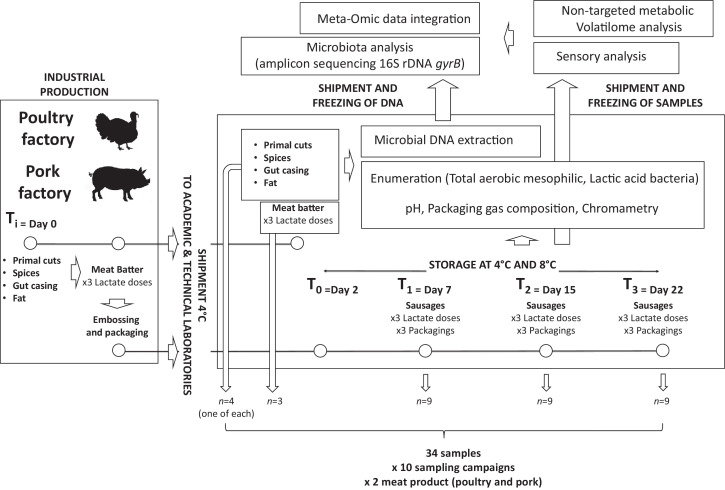
Large-scale strategy for the analysis of meat sausage samples and production of the dataset.

For industrial meat producers, predicting accurate used-by-dates for their products based on spoilage occurrence remains a big challenge. The lack of large-scale multivariate data generated and associated with specific meat productions is one of the gaps that needs to be filled before such challenge could be overcome. The objective of this project was thus to provide such dataset based on a large collaborative project between academic partners, technical centers, and two industrial producers. We collected over a six months production period, much comprehensive information on the dynamics of bacterial communities and physico-chemical variables associated with meat spoilage along the processing steps. Processed pork and poultry meat (sausages) were chosen as two examples of spoilage sensitive meat products that are among the most consumed in France. Products included all sausages ingredients (meat cuts, gut casing, spice mixes, and fat) to cover the whole meat process. The experimental work consisted in determining the diversity and the dynamics of bacterial communities during food processing and storage, and correlating them with spoilage occurrence. In the frame of this project, sensory and non-targeted metabolic volatilome analyses were performed in order to characterize the meat product spoilage in relation with food processing factors like concentration of chemical preservatives (potassium lactate) and gas composition of storage packaging. The originality of this project was to integrate biotic (microbial ecology), abiotic (sensory attributes and storage conditions) and temporal factors (monitoring along processing steps) to generate heterogeneous multivariate data for used-by-date predictive mathematical models. Fig. 1 illustrates the global experimental design of this study. Links to supplementary data (available as spreadsheets) presented in Table 1 include all the metadata characterizing each sample, as well as the samples that were selected for the different analyses (16S rDNA sequencing, gyrB sequencing, volatilome and sensorial profile).

**Table 1:** List of supplementary tables (Tables available as spreadsheets at https://doi.org/10.15454/UDQLGE) making up the dataset.

Table S1	Sample nomenclature and metadata associated with each sample.
Table S2	pH dynamics over time for the different meat products.
Table S3	Chromametric measures for the different meat products.
Table S4	Gas composition (%) of the packaging atmosphere over time for the different meat products
Table S5	Total mesophilic aerobic flora (log_10_ CFU.*g*^−1^) over time for the different meat products
Table S6	Lactic acid bacteria (log_10_ CFU.*g*^−1^) over time for the different meat products
Table S7	Non-targeted metabolic volatilome composition (reduced centered normalization of pic areas) over time for the different meat products
Table S8	Sensorial profiles over time for the different meat products
Table S9	Microbial diversity analysis based on 16S rDNA V3-V4 region amplicon sequencing including OTU abundance table, OTU taxonomic assignment table and samples metadata table usable for phyloseq R package analysis [4].
Table S10	Microbial diversity analysis based on gyrB amplicon sequencing including OTU abundance table, OTU taxonomic assignment table and samples metadata table usable for phyloseq R package analysis [4].

## 2 Experimental design, materials and methods

### 2.1 Experimental design and sampling

The project focused on the monitoring of two food matrices: pork sausages and poultry sausages. For pork sausages, two types of meat pieces were used: normal mid shoulder meat and defatted/boneless/derinded shoulder meat (also named shoulder 4D). For poultry sausage, the meat was from turkey. In both sausages, pork fat was added to a final content of 20% and 11% in pork and poultry sausages, respectively. The following additives were added in pork sausages: potassium lactate (1.13% w/w, corresponding to full normal dose); sodium acetate 0.27% (w/w); sodium ascorbate 0.06% (w/w). In poultry sausages the following additives were added: potassium lactate (2.0% w/w, corresponding to full normal dose). Furthermore, both sausages were battered with a spice mix as ingredient added to a concentration of ∼2.5% and containing (sodium salt ∼40%, dextrose ∼10%, spices ∼15%, aromas ∼22%). Ten sampling campaigns were conducted during 6 months on the production chains of two sausage producers in order to get a sufficient number of independent biological replicates. For each campaign and each food matrix, identical sampling strategy was applied. Four types of samples corresponding to the ingredients constituting sausages were collected: primal cuts, gut casing, fat and spices.

Three meat batters were separately prepared with different doses of lactate (the normal dose routinely used by the industrials, half of this dose and without any lactate). The three meat batters were sampled before their embossing into the tubular gut casing. Pork sausages were packed immediately after embossing whereas poultry sausages were packed 2 days later. Sausages were separately packaged by five into trays under three different atmospheres (normal air; 50%CO_2_-50%N_2_; and 70%O_2_-30%N_2_), sealed with thin high barrier polyester-based film PET/EVOH (Co-polymer of Ethylene and Vinyl alcohol)/PE (PolyEthylene) and stored during the first 5 days at 4 °C and then until the end of the incubation at 8 °C. The physical properties of the packaging were as follows: oxygen transmission rate <5 cm^3^/m^2^·24 *h* ^−^ ^1^·bar^−1^, CO_2_ transmission rate <25 cm^3^/m^2^. During storage, sausages were sampled at three different dates: day 7, day 15, and day 22 (which was considered as an abused storage time).

Trays were frozen at −20 °C prior color, sensory, and VOC analyses. Other treatments or analyses were performed directly.

### 2.2 Physico-chemical analyses

For all samples pH measures were performed on three sausages per tray using FiveGo FG2 with the electrode LE427-S7 (Mettler toledo, USA) inserted in sausages. For pork sausages gas composition of each tray was assessed with CheckMate3 (Dansensor, France). The same procedure was applied for poultry sausages with a digital O_2_/CO_2_ Oxybaby analyzer (WITT, Germany). Visual color changes were evaluated in triplicates for each condition, each sampling time and each production batch using the Minolta CR400 ChromaMeter (Grosseron, France) in CIE-Lab scale. The measurements determined chromatic coordinates of L* (brightness), a* (green-red balance) and b* (blue-yellow balance).

### 2.3 Bacterial collection

Ten to fifty grams of each food-product batch were mixed in a 400 ml stomacher bag of 280 µm (Interscience BagPage, France) with 4 vol (40 to 200 mL) of BK018HA peptone water (Biokar Diagnostics, France) supplemented with 1% V/V Tween 80 (VWR Chemicals, France). Mixes were treated for 3 min (pork) or 2 min (poultry) in a Masticator Homogenizator (IUL, Spain). Then, 32 mL of the shreds were collected and centrifuged at 500 × *g* for 3 min at 4 °C to spin down the food matrix fibers and debris. The still-turbid supernatant (∼25 mL) was collected and centrifuged at 3000 × *g* (pork) or 10,000 x g (poultry) for 5 min at 4 °C to spin down the bacterial cells. The bacterial pellet thus obtained was washed in 1 mL of sterile ultrapure water and collected after centrifugation as above for 5 min at 4 °C to serve directly for DNA extraction or for plating.

## 3 Plating

Bacterial counts were estimated from filtrates obtained after the stomaching step and following the ISO 4833-1:2013 and ISO 15214:1998 methods. Serial dilutions in peptone water were performed and plated on Plate Count Agar (PCA) (Oxoid, France) for pork, (Biomerieux, France) for poultry and on de Man, Rogosa, and Sharpe (MRS) agar (Oxoid, France) for pork, (Biomerieux, France) for poultry and incubated aerobically for 48 h at 30 °C to estimate the total aerobic mesophilic population and the mesophilic lactic acid bacteria population size in CFU *g*^−1^ of meat, respectively.

### 3.1 DNA extraction, amplification and sequencing

Total DNA was extracted from bacterial pellets obtained after stomaching and a centrifugation steps using DNeasy PowerFood Microbial Kit (Mobio Laboratories, USA) according to the manufacturer's instructions and as described in Poirier et al. [5]. DNA extracts were used for the amplification of the bacterial hypervariable region V3–V4 of the 16S rRNA gene with the primers V3F (5′-ACGGRAGGCWGCAGT-3′) and V4R (5′-TACCAGGGTATCTAATCCT-3′). In parallel, the degenerated primers F64 (5′-MGNCCNGSNATGTAYATHGG-3′) and R353 (5′-CNCCRTGNARDCCDCCNGA-3′) were used to amplify a ∼280-bp region of gyrB gene as described in Poirier et al. [5]. Sequencing was performed by Genoscreen (France) on Illumina Miseq sequencer using the MiSeq Reagent Kit v3 (2 × 250 bp paired-end reads).

### 3.2 Sequence read processing, OTU clustering and taxonomic assignments

Paired-end sequences were merged and trimmed as described previously [5]. Data were subsequently imported into the FROGS (Find Rapidly OTUs with Galaxy Solution) pipeline [6] to be cleaned, filtered, clustered into Operational Taxonomic units and taxonomically assigned using the Silva 128 SSU database [7] and a homemade gyrB database [5].

### 3.3 Sensory descriptive analysis

Samples of poultry and pork sausages, previously stored at −20 °C, were one-night defrost in a cold room. Just before analysis, about 50 g of sample were transferred into a 250 mL opaque glass vial and sealed with a glass stopper. A sensory panel of experts in profiling techniques (*n* = 15; at least 5 previous experiences on food sensory analysis with profiling techniques) were trained during 8 months (minimum 15 h) on altered pork and poultry sausages. During training, they developed a consensus vocabulary in order to separate important attributes describing altered sausages off-odor compared to non-altered ones. Same attributes were chosen for pork and poultry sausages. Quantitative descriptive analysis (QDA) [8] was performed using those seven olfactory attributes. The different attributes and their respective descriptors used for the evaluation of the samples were global alteration odor, rancid, eggy/sulfurous, ethereal/fermented fruit, fermented/old dry sausage, old cheese, sour/pungent. The QDA was performed in ten sessions. Each panelist received randomly 12 coded samples in a session. Samples were presented monadically according to a balanced design in vials containing sausage for sniffing. Sensory evaluation was carried out at room temperature (25 ± 2 °C) in isolated booths in a sensory lab. Each panelist rated the global alteration odor intensity and the 6 odor attributes intensities of each sample on a 10 cm unstructured line scale (from “absent” to “very intense”) with sensory evaluation software (Fizz Biosystems, France).

### 3.4 Volatile organic compound (VOC) analysis

Samples of poultry and pork sausages, previously stored at −20 °C, were cut in small pieces in a cold room to minimize the volatilization of VOC. Around 3–4 g of sample were transferred into a 20 mL headspace vial and sealed with a screwcap with silicone rubber septum. The vial was weighted before and after sampling to determine the exact weight of sausage. Analyses were carried out in triplicate. VOC were determined by HS–GC–MS. All analyses were performed on a Varian 450 gas chromatograph (Varian, USA) coupled to a Varian 225-IT mass spectrometer (Varian), equipped with a CTC Combi PAL (CTC Analytics AG, Switzerland). Samples were equilibrated by agitation at 60 °C for 20 min prior to injection and 1 mL was drawled from the headspace to inject in the GC. The HS-GC–MS conditions were as follows: capillary column: DB-624 UI (30 m x 0.25 mm I.D × 1.4 mm film thickness) (Agilent Technologies, USA); Carrier gas: Helium with a flow rate of 1.4 mL.min^−1^; Injection port mode: splitless; Needle temperature: 60 °C; Injection temperature: 220 °C. The oven temperature was programmed from an initial temperature of 40 °C (7 min holding), rising to 50 °C at 4 °C/min (1 min holding), to 70 °C at 4 °C/min (1 min holding), to 120 °C at 3 °C/min (2 min holding) and to 245 °C at 30 °C/min (4 min holding). Transfer line temperature: 250 °C. The temperatures of the manifold and the ion trap were kept constant at 150 °C and 40 °C respectively. Data were obtained in scan mode at four scans/s in the mass range (*m/z*) of 35–350 atomic mass units. VOC were identified by comparison of GC retention times and mass spectra with those of the standard compounds. Peak area (in UA) was used as quantitative data to monitor the relative changes of VOC over storage, potassium lactate concentration and atmosphere packaging. Data were initially subjected to pre-processing using the standard normal variate transformation to facilitate comparison of the peaks with different magnitudes.

## Conflict of Interest

The authors declare that they have no known competing financial interests or personal relationships which have, or could be perceived to have, influenced the work reported in this article. This work was supported by the ANR RedLosses Project, Grant ANR-16-CE21-0006, operated by the French Agence Nationale de la Recherche. SP and NDML were granted for postdoctoral and PhD study respectively through this agency. The funding body did not play any role neither in the design of the study nor in collection, analysis, and interpretation of data.

## Appendix Supplementary materials

Image, application 1

Image, application 2

Image, application 3

Image, application 4

Image, application 5

Image, application 6

Image, application 7

Image, application 8

Image, application 9

Image, application 10

Image, application 11
